# Epidermal mTORC1 Signaling Contributes to the Pathogenesis of Psoriasis and Could Serve as a Therapeutic Target

**DOI:** 10.3389/fimmu.2018.02786

**Published:** 2018-11-30

**Authors:** Claudia Buerger

**Affiliations:** Department of Dermatology, Venerology and Allergology, Clinic of the Goethe University, Frankfurt am Main, Germany

**Keywords:** psoriasis, mTORC1, keratinocytes, rapamycin, topical agent

## Abstract

Although modern biologics targeting different inflammatory mediators show promising therapeutic success, comprehensive knowledge about the molecular events in psoriatic keratinocytes that contribute to the pathogenesis and could serve as therapeutic targets is still scarce. However, recent efforts to understand the deregulated signal transduction pathways have led to the development of small molecule inhibitors e.g., tofacitinib targeting the Jak/Stat cascade that opens additional therapeutic options. Recently, the PI3-K/Akt/mTOR signaling pathway has emerged as an important player in the control of epidermal homeostasis. This review summarizes the current knowledge on the role of this pathway in the pathogenesis of psoriasis, especially the epidermal manifestation of the disease and discusses current approaches to target the pathway therapeutically.

## Introduction

Psoriasis is a common, chronic inflammatory skin disease that affects 2–3% of the population and is associated with a reduced quality of life and a shortened life expectancy due to the association with the metabolic syndrome and cardiovascular pathologies (1). Clinically psoriasis presents with red, scaly plaques, which mostly affect predilection sites such as extensor surfaces of forearms and shins, umbilical, perianal, retro-auricular regions, and scalp (2). These plaques are characterized by epidermal hyperproliferation with impaired keratinocyte differentiation, extravasation of lymphocytes, and angio(neo)genesis. Currently it is assumed that sustained activation of plasmacytoid dendritic cells by epidermal antigens due to skin trauma or infection is the first step in the pathogenesis of psoriasis (3). This induces the maturation of myeloid dendritic cells, which in turn promote via secretion of IL-6, IL-12, and IL-23 the differentiation of T cells into Th1 and Th17 cells (4). Their effector cytokines such as IL-17, IL-22, and TNF-α induce and maintain hallmarks of psoriasis such as keratinocyte proliferation, and disturbed differentiation, leading to epidermal acanthosis, hyperkeratosis, and parakeratosis (5). Activated keratinocytes in turn produce important proinflammatory cytokines and chemokines that are able to recruit a broad spectrum of inflammatory cells from the vascular system. Thus, a “vicious circle” of excessive immune response, epidermal hyperproliferation, and neovascularization is initiated, which leads to the complex clinical appearance of psoriasis (6). The immunological events leading to the described epidermal changes are well understood and various “biologics” against different inflammatory cytokines such as TNF-α, IL-17A, or IL-12/IL-23 show promising results in the therapy of psoriasis (7). However, comprehensive knowledge about the intracellular epidermal processes induced by the immunological network, and which could serve as potential therapeutic targets, is still missing. There is some evidence that signaling pathways such as Stat1, Stat2, and Stat3 (8–10), MAPK family kinases (11–14), Wnt5a (15), or NF-kB (16–20) are dysregulated in the psoriatic epidermis and some of them have been targeted by molecular inhibitors (21, 22).

## The PI3-K/Akt/mTOR signaling cascade

The serine/threonine kinase Akt, also known as protein kinase B (PKB), represents a crucial signaling point in eukaryotic cells and plays a central role in the regulation of cellular processes such as growth, proliferation, and metabolism (23). One of the main downstream mediators of Akt is the mTOR signaling pathway. mTOR (mechanistic target of rapamycin) occurs in two different multiprotein complexes, both of which possess the mTOR kinase as a catalytic subunit and share some regulatory proteins (mLST8, Deptor), while other proteins are complex-specific. Specific to the mTOR complex 1 (mTORC1) is the scaffold protein Raptor, which regulates the assembly and localization of the complex. This complex can be inhibited by rapamycin (24). The rapamycin-insensitive mTOR complex 2 (mTORC2), on the other hand, additionally consists of the scaffold proteins Rictor and Protor1/2 and phosphorylates Akt on Ser473 (25) and thus regulates proliferation and cell growth.

**Figure 1:**
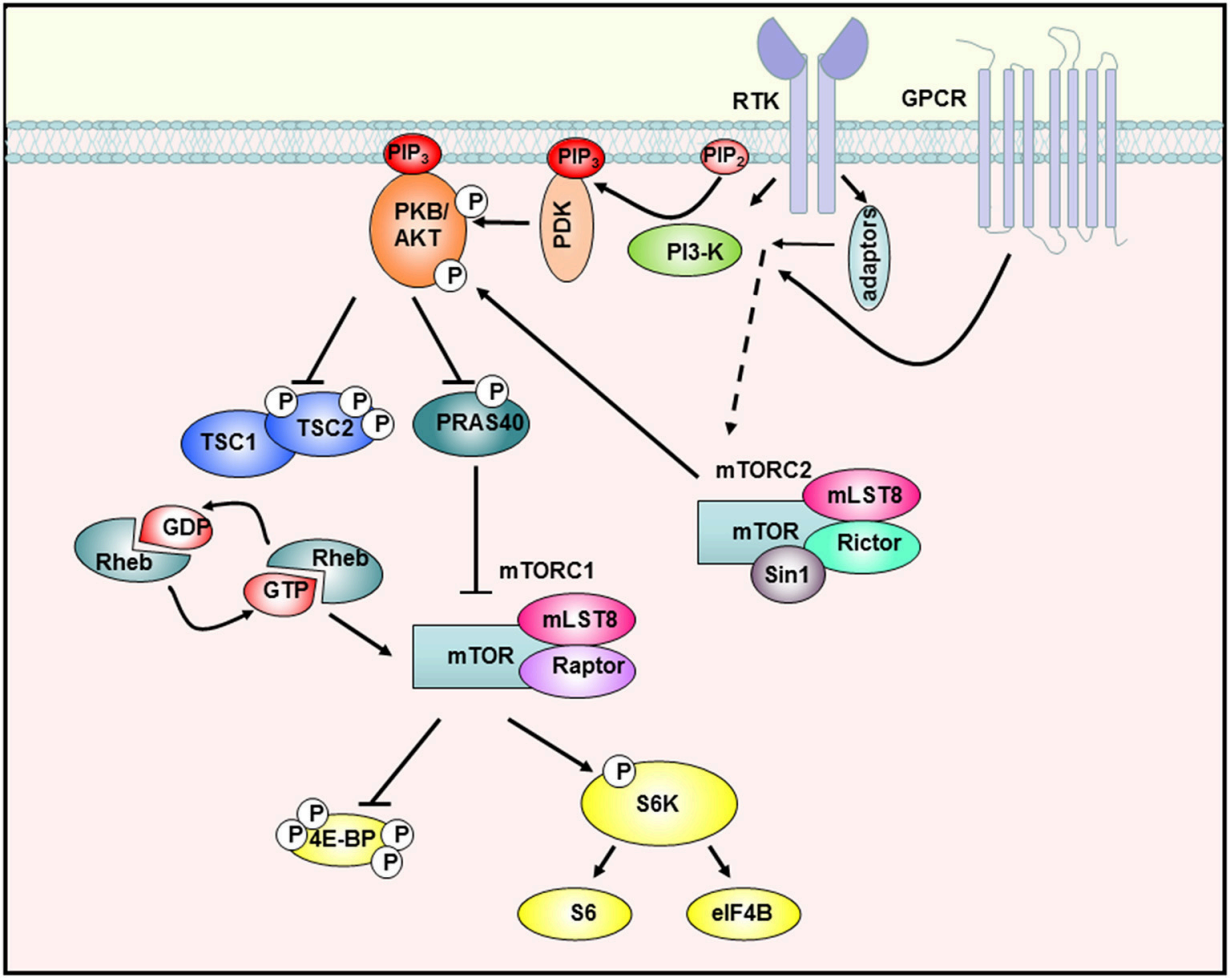
The PI3-K/Akt/mTOR signaling cascade. Stimulation of receptor tyrosine kinases (RTK) and G-protein-coupled receptors (GPCR) leads to activation of PI3-K, which then synthesizes PIP_3_ in the membrane. Subsequently, Akt is recruited to the membrane and phosphorylated by PDK-1 and mTORC2. The activated Akt kinase phosphorylates various substrates such as PRAS40, which is then inactivated and releases mTORC1. In addition, TSC2 is inhibited so that the downstream GTPase Rheb remains GTP-bound and can activate mTORC1. The fully activated mTORC1 complex then activates proteins of the translation machinery by phosphorylating S6K-1 or 4E-BP1 [adapted from Manning et al. 23).

After ligand binding to cognate receptors such as tyrosine kinase receptors (RTK) or G-protein-coupled receptors (GPCR) phosphatidylinositol 3-kinase (PI3-K) becomes activated either directly or via adaptor proteins like the insulin receptor substrate 1 (IRS-1) (Figure 1). PI3-K mediates the synthesis of 3′-phosphoinositides (PIP_3_) at the plasma membrane, which act as lipid-second messengers and recruit Akt and the phosphoinositol-dependent-kinase (PDK1) to the membrane. PDK1 can then activate Akt by phosphorylation on Thr308. For complete activation of Akt, phosphorylation of Ser473 by mTORC2 is required. Fully activated Akt is then able to phosphorylate a large number of signal molecules with different functions in the control of growth, proliferation, metabolism, or apoptosis (26). Akt and other signal molecules regulate the TSC complex, consisting of TSC1 and 2 (Figure 1). This complex is an important regulator of mTOR by acting as a GTPase-activating protein for Rheb (Ras homolog enriched in brain). The GTP-bound form of Rheb interacts directly with mTOR and activates the complex (27, 28). Furthermore, Akt phosphorylates the proline-rich Akt substrate of 40 kDa (PRAS40), whose inhibitory interaction with mTOR is then dissolved (29), so that the mTOR kinase is fully activated.

By phosphorylation of downstream molecules, mTORC1 regulates the biosynthesis of macromolecules necessary for cellular growth and proliferation. By phosphorylating two key proteins of translation initiation S6 kinase-1 (S6K-1) and eukaryotic initiation factor 4E (eIF-4E) binding protein-1 (4E-BP1), mTORC1 controls the rate of protein biosynthesis (30). In particular, mTORC1 regulates the translation of mRNAs with a so-called 5′TOP (5'terminal oligopyrimidine) motifs. These mRNAs mainly code for ribosomal proteins and components of the translation machinery (31), so that mTORC1 activity also contributes to the general synthesis of proteins in this way. Furthermore, mTORC1 controls the synthesis of lipids through regulation of the transcription factor SREBP (32), the production of nucleotides (33), and inhibits catabolic processes such as autophagy (34).

## mTOR signaling in psoriasis

Recently attention has been drawn to the PI3-K/Akt/mTORC1 cascade as a regulator of epidermal homeostasis and its putative role in inflammatory skin diseases. Akt is highly activated in all epidermal layers of psoriatic lesions (35), except the basal Ki-67 positive layer that represents dividing cells (36). This may be explained either by psoriatic keratinocytes that keep their proliferative pathways turned on, even after leaving the basal layer. Alternatively, Akt could prevent cellular apoptosis, which also contributes to the fast maturation process of psoriatic keratinocytes (37). Inhibition of PI-3K/Akt could be a promising therapeutic strategy as the Vitamin D analog 1α, 25-dihydroxyvitamin D3-3-bromoacetate (BE) reversed IL-22-induced psoriasiform changes *in vitro* (38).

**Figure 2:**
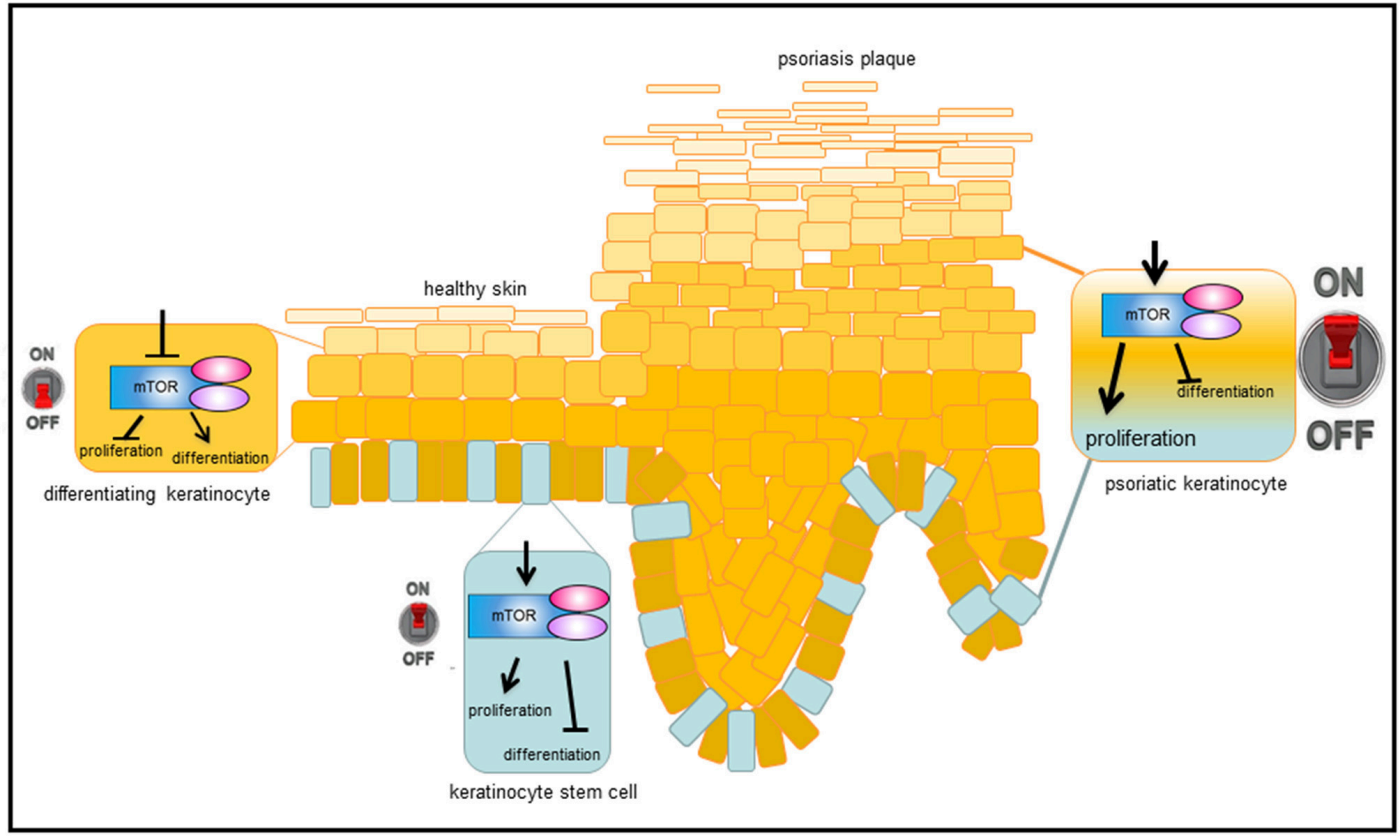
Model, how mTORC1 signaling controls epidermal homeostasis and contributes to psoriasis. In healthy, basal keratinocytes, mTORC1 signaling pathway is active and controls proliferation while blocking differentiation. When cells leave the proliferative, basal compartment, mTORC1 is switched off and differentiation is enabled. Under inflammatory conditions, such as in psoriasis, mTORC1 is permanently activated, which leads to massive proliferation in the basal layer and disturbed keratinocyte differentiation in suprabasal layers, resulting in the phenotypic changes typical of psoriasis.

Our group showed for the first time that the central mediator of Akt signaling, the kinase mTOR, is hyperactivated in lesional and nonlesional skin of psoriasis patients, while downstream signaling molecules such as S6K-1, the ribosomal protein S6, and 4E-BP1 are only activated in suprabasal layers in lesional skin (39, 40). Furthermore, it was shown that additional components of mTORC1 such as Rheb and Raptor are overexpressed in psoriatic skin and others such as PRAS40 are hyperactivated (40). That hyperactivated mTORC1 signaling is indeed an important aspect in psoriasis, showed the work by Shirsath et al.: In a genetic mouse model of psoriasis, PUVA treatment not only ameliorated the histological psoriasis score, but also normalized mTORC1 signaling (41). The divergent localization of the activated signal components in the epidermis points toward a pathophysiological contribution of deregulated mTORC1 signaling in psoriasis. For example, the hyperactivation of mTORC1 in the basal layer may indicate a role during the enhanced proliferation of psoriatic keratinocytes, while the suprabasal hyperactivation points toward a role in aberrant differentiation. Using different *in vitro* approaches our group showed that healthy keratinocytes switch off Akt/mTORC1 signaling as soon as differentiation is initiated. This appears to be associated with proliferation control, as Ki-67 positive cells in the basal layer of healthy skin also showed mTOR activity. Thus, inactivation of mTOR seems to be a prerequisite for keratinocytes to initiate terminal differentiation. In contrast, in an inflammatory environment such as psoriasis, the mTORC1 cascade is aberrantly activated in all epidermal layers. We were able to show that IL-1β, IL-17A, TNF-α, and in particular a mix of these cytokines leads to activation of the mTORC1 signaling cascade. In addition, the pathway might be activated by miRNAs that are deregulated in psoriatic skin (42, 43), or by mechanosensitive molecules such as polycysteins (44). The latter could explain the predilection of psoriatic plaques to sites of increased mechanical stress such as elbows and knees. Our group could prove that continuous mTORC1 activity contributes to the proliferation of keratinocytes and simultaneously inhibits proper keratinocyte maturation. Thus, we suggest a model where mTORC1 signal transduction functions as a central switch between keratinocyte proliferation and differentiation (Figure 2). This model is supported by findings from Mitra et al. showing that IL-22 regulates keratinocyte proliferation via the Akt/mTOR cascade (45).

Apart from this model also other mechanisms, how epidermal mTORC hyperactivation can contribute to the pathogenesis of psoriasis are being discussed. Patel et al. could show that the release of pro-inflammatory mediators such as IL-6, CXCL8, or VEGF by keratinocytes is mediated via mTORC (46). Another mechanism by which hyperactive PI3-K/Akt/mTORC1 signaling might contribute to the pathogenesis of psoriasis could be through inhibiting autophagy (47). Autophagy and more specifically nucleophagy is an important mechanism during keratinocyte differentiation and maturation into corneocytes. Thus, high mTORC1 activity inhibits nuclear degradation and contributes to parakeratosis (retention of nuclei), one of the hallmarks of psoriasis (48).

Although not the main focus of this review, it has to be mentioned that mTORC1 signaling also has important functions in the innate (49) and adaptive immune system (50–52). Specifically a role for mTORC1 and 2 has been attributed to the regulation of immune cell energy metabolism and thereby to the control of their function and differentiation (53). Deregulated mTORC1 signaling was found in peripheral blood mononuclear cells (PBMCs) of psoriasis patients (54), which seems to contribute to their pathological behavior (55). Regulatory T-cells from psoriasis patients show increased mTOR phosphorylation and treatment with methotrexate reduces mTOR activation (56). In addition, a novel vitamin D analog reduced mTORC1 activity in activated memory T cells form psoriasis patients and thus contributed to the immunosuppressive effect of the drug (57).

## mTOR signaling as a therapeutic target in psoriasis

The mTOR complex is also interesting because of its inhibitor rapamycin (sirolimus), which was isolated from Streptomyces hygroscopicus in 1975 (58). This bacterial strain was first found in the soil of Rapa Nui Island (Easter Island), after which the substance was named. Even before the kinase mTOR was identified as a target protein of rapamycin in 1994 (59), rapamycin was known for its anti-proliferative properties on lymphoid cells and associated immunosuppressive properties (60). Rapamycin is therefore still used to prevent transplant rejection (61) and restenosis after implantation of stents in coronary vessels (62). In addition, anti-tumor effects of rapamycin and its analogs (rapalogs) have been under investigation (63, 64).

It is particularly interesting that rapamycin has also been tested for its antiproliferative and immunosuppressive properties in a few small studies in psoriasis patients. Systemic administration of everolimus (a derivative of sirolimus) was successful in a single patient (65), whereas a larger study showed good results for sirolimus in combination with cylosporin therapy (66). In addition, in a renal transplant patient with refractory psoriasis, everolimus ameliorated skin lesions (67). Remarkably, only limited new substances for topical anti-psoriatic therapy have been developed in recent years and new product launches mostly consisted of derivatives or further developments of established agents (68). Thus, the establishment of new substances for topical application is desirable. In one small trial topical treatment with rapamycin led to a significant improvement of the clinical score, while the thickness of the plaques was unchanged (69). To further explore this therapeutic option, the effectiveness of topical rapamycin was investigated in the imiquimod-induced psoriasis mouse model, which showed activation mTORC1 signaling similar to human psoriasis (70, 71). Mice treated with rapamycin showed a significant improvement in clinical appearance (redness, swelling, and flaking), reduced angioneogenesis and normalization of epidermal thickness compared to the control group. While the imiquimod-treated mice showed a clear activation of mTORC1 and downstream molecules, rapamycin reduced the activity to the level of untreated mice. Rapamycin normalized the expression and distribution of differentiation markers such as keratins, involucrin, and loricrin. In addition, the influx of innate immune cells into the draining lymph nodes was partially reduced by rapamycin treatment (71). In the same mouse model rapamycin treatment also restored the expression of tropomyosins, which are downregulated in psoriatic lesion and could also contribute to the disease (72).

Rapamycin is an allosteric inhibitor, that requires binding to its intracellular receptor, FKBP12, to selectively inhibit some, but not all functions of mTORC1 (73). mTORC2 is considered rapamycin-insensitive, although it can be inhibited by chronic rapamycin treatment in some cell types (74). To inhibit all functions of both complexes, selective ATP-competitive inhibitors of mTOR were developed (75, 76). As they are efficiently inhibiting both mTOR complexes and thus inhibit Akt signaling, they could be interesting therapeutic compounds in psoriasis. The same rationale was applied, by Chamcheu et al., that showed efficient inhibition of PI3-K, mTOR, and S6K-1 by Delphinidin, an antioxidant plant pigment (77). Topical Delphinidin was able to ameliorate symptoms in two different psoriasiform mouse models (77, 78).

In summary, there is increasing evidence that specifically topical application of mTORC inhibitors can be a successful strategy for anti-psoriatic therapies and underline the need to further explore the mTORC1 signaling pathway as a therapeutic target in psoriasis.

## Author contributions

CB conceptualized and wrote the manuscript and created the figures.

### Conflict of interest statement

The author declares that the research was conducted in the absence of any commercial or financial relationships that could be construed as a potential conflict of interest.
